# Colloidal and Sedimentation Behavior of Kaolinite Suspension in Presence of Non-Ionic Polyacrylamide (PAM)

**DOI:** 10.3390/gels8120807

**Published:** 2022-12-09

**Authors:** Aref Abbasi Moud

**Affiliations:** Department of Chemical and Biological Engineering, The University of British Columbia, Vancouver, BC V6T 1Z3, Canada; aabbasim@ucalgary.ca

**Keywords:** kaolinite particles, colloids, polyacrylamide, maximum packing, sedimentation, LUMisizer

## Abstract

Colloidal behavior of kaolinite particles in water was investigated in this manuscript, without and with the addition of a polymer flocculant (non-anionic polyacrylamide (PAM)), using diverse imaging techniques in addition to LUMisizer. The addition of PAM was found to be causing the formation of bridges among particles thus increasing their settling rates to the bottom of the container. To assess the size of flocs and the potential morphology of PAM around particles and their clusters, the state of flocs formation and polymer distribution was analyzed through various microscopical techniques, namely scanning electron microscopy (SEM) and transmission electron microscopy (TEM). SEM and TEM results revealed that, in the absence of PAM, the floc structure of the sediment was loose and irregularly distributed, while the presence of PAM made the sediment structures greatly denser. Later, using LUMisizer, dynamic light scattering (DLS) and the zeta potential of kaolinite, sedimentation, and colloidal behavior of suspension came under scrutiny. Using LUMisizer, the maximum packing and settling rates of the particles were experimentally obtained as roughly 44 vol%; settling rates were estimated in 63–352 µm/s when centrifugal force varied and, using maximum packing values, compressive yield was estimated to vary between 48–94 kPa. The results of this study are instructive in choosing appropriate polymers and operating conditions to settle clay minerals in tailing ponds. Additionally, the maximum packing of kaolinite particles was simulated with spherical particles with varied polydispersity to connect DLS data to the maximum packing values obtained using LUMisizer; the little discrepancy between simulation and experimental values was found to be encouraging.

## 1. Introduction

Clay minerals are everywhere in most mine tailings and wastewater ponds. They create numerous problems in the transport of tailings effluents and affect the stability of tailing ponds over a long course of time [1,2,3]. One source of the problem is the surface chemistry of clays; the chemistry of the interface is usually heterogeneous [4]; it has different charges (positive and negative) on the basal faces and the edges of the clay layers. The basal face usually has a pH-independent negative charge, while edges have sites dependent on the pH of the medium [5]; this development is an origin of the development of different morphologies of attachment, i.e., edge-to-edge, edge-to-face, or face-to-face aggregate organizations, in the absence of coagulant. Fine clays, such as kaolinite, have huge implications on the management of effluents in copious industries; therefore, it is critical to facilitate their settlements in tailing ponds through the adoption of proven strategies. Facilitation of clay sedimentation requires an understanding of its settling behavior [6,7]. 

Contrary to micron-sized inclusions, the colloidal behavior of kaolinite suffers from a sluggish settlement pace over time as particles are small and hydrodynamic forces, electrostatic and van der Waals interactions, are not negligible. Hence, proven strategies that can facilitate settlement are required. One route is to use polymer flocculants; the addition of a polymer coagulant facilitates settlement through polymer bridging, charge neutralization, and, in some cases, complex formation between particle surfaces and polymer molecules, or a combination of these [8]. 

The mechanism of adsorption of polymer onto particle is as important as the selection of the simple system for flocculation. Non-anionic polyacrylamide (PAM), as a polymer flocculant, has been observed to bridge clay particles efficiently in the literature [8]. The reports of Mpfou et al. [9] and Lee et al. [10] indicate that interactions, and the following adsorption, between kaolinite particles and PAM chains materialize via hydrogen bonding, hydrophobic interactions, and ion-dipole interactions. The adsorption process of PAM chains on kaolinite takes place via the following mechanisms: reptation of polymer chains inside the medium toward the interface, linkage of polymer segments, and relaxation or reorientation of polymer chains while in bond at the interface. There is a recent review article published in the area by our group that has captured most of the discussion on the interplay between polymer addition and particles present in the system [8,11]. 

Through the lens of experiments, to measure particle packing, a receptacle is filled with many uniform balls, which are then shaken down and measured for volume. The number of balls present, and the measured value of each ball’s diameter, can be used to determine the intrinsic volume of the spheres. In addition to the fundamental query of the packing’s randomness, there are two significant experimental challenges. One is the propensity for some sort of almost-regular packing to occur at the vessel’s walls, especially when those walls are even remotely plane. The additional vacant space at the boundary is the other. Since the two effects’ mistakes are in the opposite direction, a simple experiment can produce findings that are quite close to those of more complex observations. The packing with the lowest density is known as “loose” packing, while the packing with the highest density is known as random tight packing. A packing density that falls between these two boundaries is often obtained by dropping a group of uniform balls into a container. Therefore, in addition to challenges rife within experiments, these endeavors are also time-consuming. 

In addition to polydispersity, maximum packing is also influenced by the form of the particles. According to the simulation in ref. [12], the order of basic three-dimensional random packing densities, from highest to lowest, is cube > ellipsoid > cylinder > sphero-cylinder > tetrahedron > cone > sphere, whereas the order of ordered packing densities, which represents the densest possible arrangement of parts, is cube > cylinder and spherocylinder > cone > tetrahedron > ellipsoid > sphere [12]. The two orders, orderly packing and disordered packing, are very unlike one another. It follows that varied packing density estimates will likely result from using particles with varying shapes in both theory and simulation. 

In the literature, reports suggest that it has been difficult to define and determine the random close packing (RCP) limit for frictionless hard-sphere particles theoretically; however, due to easier computational access and the development of more efficient algorithms, the process of estimation of random close packing has become more efficient and less challenging. There are at least three estimates for the RCP limit of monodisperse particles, each with a different density: (i) φ, φ = 0.634–0.636 [13,14,15,16]; (ii) 0.64 [17,18,19,20]; and (iii) 0.65 [21,22,23,24,25,26,27]. Even though these values closely follow one another, the values of 0.634 and 0.65 are supported theoretically [26]. In another report [28], authors demonstrated that the values of 0.64 and 0.65, which correspond to the RCP limit and a lower bound of the glass close packing (GCP) limit, respectively, belong to separate occurrences [29].

In this study, the flocculation behavior of kaolinite particles in a water medium in the absence and presence of high molecular weight PAM was investigated, and the relationship between the settling rate of kaolinite particles and polymer flocculant dosage was investigated. This analysis was also extended to the packing density of particles and particle sizes post-flocculation. The impact of PAM on the development of the kaolinite floc microstructure was seen using scanning and transmission electron microscopy (SEM, TEM). The comprehension of kaolinite floc formation in the presence of a non-ionic polyacrylamide flocculant was improved by such a comprehensive visualization. The optimal polymers and operating conditions for the flocculation of clay minerals in tailing ponds may therefore be chosen using this knowledge. 

Later, data on settling rates, particle size, and maximum packing values that can aid in the manufacture and handling of suspensions were acquired using LUMisizer. The maximum packing of kaolinite particles modeled as spheres was simulated using simulations that adhered to forced-biased algorithms (FB) and Lubachevsky–Stillinger (LS). According to the results, algorithms need to be improved to accurately capture the arrangement behavior of kaolinite suspensions following sedimentation. The effectiveness of simulation in predicting the right maximum packing based on the polydispersity of kaolinite particles ascertained from DLS data was tested using these simulation-based experimental data on maximum packing. With the combined strength of innovative colloidal behavior analysis and simulation, the hybrid technique of the current publication facilitates the understanding of the formulation of futuristic flocculants for accurate analysis and hastens the settling of kaolinite and other nanosized particles. 

The data in the study have been arranged as follows: First, characterization of particles for charges and sizes both alone and with flocculant was attempted. Later, to get insight into the shape of flocs and the general organization of the polymer on top of particles, later flocs under the impact of polymer were studied using SEM and TEM. Then, using LUMisizer, two sets of data—qualitative and quantitative—were produced concerning the rate of sedimentation as well as the maximum packing in the presence and absence of the flocculant, which allowed for the computation of compressive yield stress. 

Finally, using tested algorithms for simulating maximum packing, assuming kaolinite acted as hard spheres, we sought to simulate maximum packing. These algorithms were evaluated for their ability to forecast maximum packing, given polydispersity. As DLS provided the polydispersity of particles, using simulation, maximum packing could be generated and compared against experimental data. 

## 2. Experimental Section

### 2.1. Materials

Kaolinite was purchased from Sigma Aldrich. Kaolinite (A1_4_Si_4_O_10_ (OH)_8_) is a 1:1 layer clay entity, which is composed of a tetrahedral SiO_2_ plane and an octahedral AlO_2_(OH)_4_ plane connected via oxygen atoms. Along with the main particle, here designated as Sigma I, two additional kaolinite samples were obtained for initial experiments to compare their settling rates and colloidal behavior and examine their maximum packing using LUMisizer. These particles have been fully characterized in our previous publications in terms of their size and geometry, rheological, and colloidal slip behavior [2,30]. Two other kaolinite samples are labeled here on out as Sigma II and Riedel consistent with our previous publications.

Kaolinite flocs were prepared by adding 0.1, 2.5, 7, and 14 wt% kaolinite into DI water medium. The suspension was stirred at 400 rpm for 30 min and then sonicated for 20 min through on and off pulses every 10 s. The pH of the suspension was measured to be 6.8.

Sigma Aldrich was also utilized to obtain high molecular weight non-anionic polyacrylamide (PAM), which was employed as the polymer flocculant and has a molecular weight (Mw) of 5–6 million g/mol. PAM was dissolved in deionized water at a concentration of 3 weight percent by agitating the mixture for 20 min at 200 rpm. Additionally, the solution was aged for 24 h before being used. The polymer chains in the solution must fully extend during this time for hydrolysis to occur. Dropwise additions of PAM solution were added to the suspension while mixing at 400 rpm, bringing the concentrations to 0.08 weight percent, 0.1 weight percent, 0.2 weight percent, and 0.3 weight percent. It is worth noting that the percentage of acryl and amide groups in the backbone of the polymer concerning the rest of the PAM molecule determines the charge density [31]. Due to the susceptibility of the polymer solution toward degradation, a fresh solution of the polymer in de-ionized (DI) water was made every five days.

### 2.2. Transmission and Scanning Electron Microscopies

The position of PAM on kaolinite clusters was monitored using TEM while studying the floc structure of kaolinites. A Tecnai TF20 G2 FEG-TEM (FEI, Hillsboro, OR, USA) was used to take TEM images, and a 200 kV acceleration voltage was used. A droplet of the created solution, holding 5 L, was placed on a copper grid that was coated in carbon before being imaged by a TEM. Additionally, flocs received additional SEM examination. Using a scanning electron microscope, the micro-morphology of the created flocs was examined (XL30, Philips). The produced flocs were freeze-dried using liquid nitrogen prior to SEM imaging. Gold was then sputtered when a little portion of freeze-dried hydrogel was placed on a silicon wafer. 

### 2.3. Particle Size Analysis Using Dynamic Light Scattering

Dynamic light scattering was employed to gauge the particle’s size using a Malvern Nano-Zetasizer (Malvern Devices, Nano ZS, Malvern, UK) and a 632.8 nm, 4 mW light. A polystyrene (PS) cuvette was filled including about 1.4 mL of suspension (DTS0012, Malvern). For clay dispersion, the phase refractive index used was 1.55; for continuous phase, the refractive index was chosen as 1.33 and the dynamic viscosity of 8.9 × 10^−4^ [32]. As required by the zeta sizer program, 3 measurements were carried out over the course of 14 runs each. For Sigma I, the volume mean particle size averaged out to around 1 µm. Sigma I has a somewhat polydisperse particle size distribution with a single peak at the particle’s main size. 

### 2.4. Zeta Potential Analysis

Kaolinite particles dispersed in DI water were characterized for zeta potential and size using Nano-Zetasizer (Malvern Instruments, Nano ZS, Malvern, UK). For the identification of aggregates and the measuring of tiny to moderately large flocs (0.3 nm to 10 m in diameter) in diluted samples, the Zetasizer Nano ZS equipped with two analyzers was utilized. The electrophoretic mobility of the dispersion medium was estimated using the zeta sizer, and Henry’s equation was then used to calculate the zeta potential of suspension. Prior to inserting the measurement cell into the apparatus, care was taken to ensure that the sample was free of bubbles. The average zeta potential was measured five times; for Sigma I, it was −17 mV with a standard variation of 1 mV; for Sigma II, it was −20 mV with a standard deviation of 2 mV; and for Riedel, it was −24 mV with a standard deviation of 1 mV. The Riedel sample therefore seemed to be more charged than the other inclusions at neutral pH. These numbers are significant because they appear to be sufficient to maintain the particle’s stability in suspension over time, and the zeta potential values measured here closely match those published elsewhere. [30,33]. Using 0.1M NaOH or H_2_SO_4_, the pH of the suspensions was adjusted. The zeta potential of the clay samples (1 g at 20 g/L concentration) was determined in a 1.0 mM KCl solution after they had been agitated in the same manner as described in the adsorption studies section. At a constant electric field (8.4 V/cm) and room temperature, all measurements were made. 

**Figure 1:**
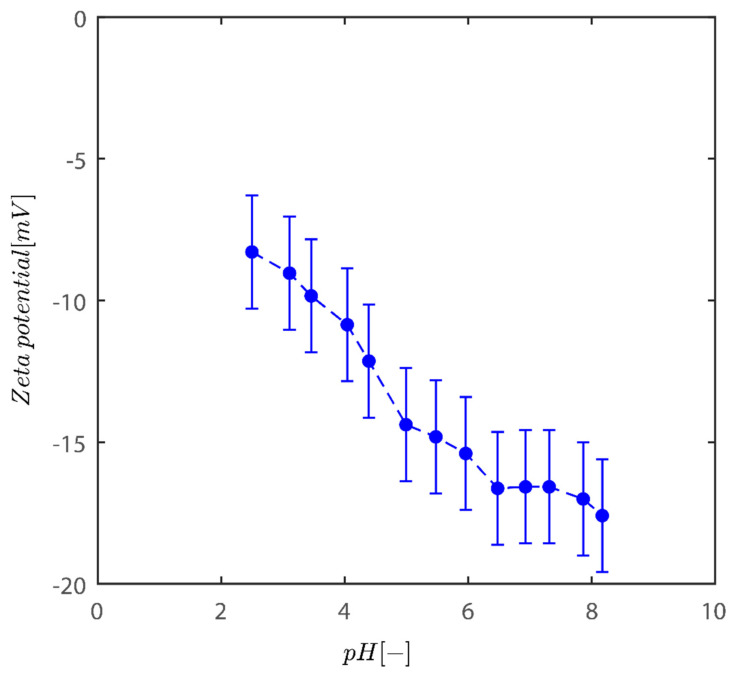
Variation of zeta potential with pH of kaolinite suspensions across pH examined here between 2 and 8.2.

The findings of the fluctuation of the zeta potential for the Sigma I sample are given in Figure 1. As the acidity of the medium diminishes, the system tends to become more negative. In addition, charges are one of the factors that influence the colloidal behavior of kaolinite particles [2,30].

Although in those described situations the zeta potential values at pH~8 reach values of −50 mV, the fluctuation in zeta potential values agrees well with data for bentonite and two additional models of kaolinite from Riedel and unimin kaolinite [34]. In the pH range of 2–8, the zeta potential is negative for kaolinite reported. This behavior is not unusual for these clays [35,36,37]. The clay particles therefore have a net negative charge over the whole pH range. In both kaolins, and to a limited extent in bentonite, the negative electrostatic potential decreases in size at low pH. This is because the permanent surface charges produced by the isomorphic substitution of smaller valency metal ions in the clay crystalline structure are gradually neutralized by the pH-dependent positive edge charges, which become more important with lowering pH.

Even at low pH levels, these positive edge charges, however, are insufficient to entirely neutralize all of the lasting negative charges [38]. One cause is the positive charge concentration being restricted by the comparatively small edge surface area. It could only account for 5% of the entire surface area [39]. Kaolinite has fixed negatively charged sites on the basal planes because of the lower positive valence ions isomorphically replacing the inner Si and Al ions in the crystalline structure. Positions reveal Si and Al OH groups in hydroxyl-terminated planes. The spots are dynamically charged, either negatively or positively, and vary based on the pH levels; positive charges may form on the alumina sides and edges as an outcome of direct H/OH- transfer from the aqueous medium.

### 2.5. LUMisizer 

Under the experimental conditions, the size distribution of flocs was determined. The volume average diameters were measured by constant position analysis. Although it is feasible to establish a link between concentration and impeded sedimentation velocity, position suspension in a very diluted concentration might make sedimentation determination challenging and error prone. Future research will thus be devoted to attempting to connect sedimentation velocity to concentration to identify the threshold over which settling rate is hampered by surrounding particles. For now, instead of scrutinizing the study of hindered velocity caused by surrounding particles, only raw data has been examined. 

Analysis of centrifugal sedimentation is done using LUMisizer. The analytical centrifugation analyzer LUMisizer (LUM GmbH, Germany) [40] was used to determine the sedimentation velocities of kaolin and KLD/kaolin foci. Each measurement involved adding 1.1 mL of a clay solution (unless otherwise stated) to the instrument’s cell, centrifuging it for 20 min at various speeds (200× *g*, 400× *g*, 500× *g*, 1000× *g*, and 4000× *g* rpm), and then collecting transmission data every 5 s. Measurements were made with a light factor of 1 and a temperature of 25 °C. 

The thickness of the sedimentation layer in the cell was plotted as a function of time for each run at various RCFs (6–2325 g), and the slope of this line provided the sedimentation velocity (µm/s) of the particles at each RCF. The size distribution of flocs was established in accordance with the experimental circumstances. Constant position analysis [41] was used to measure the volume average diameters. To verify that the findings of the analysis were typical for all measures, three points (about 115.0, 120.0, and 125.0 mm) within the detection region (103–130 mm) were selected in each measurement. 

#### 2.5.1. Sedimentation Velocity Analysis

The kaolinite particle settlement speed at 25 °C was examined using the LUMiSizer dispersion stabilization analyzer (298.15 K). To help the particulate suspensions settle at various relative centrifugal forces (RCF), which is the centrifugal relation to the force of gravity (g) that is given to such a particle, centrifugal forces were given to the fluids. Equation (1) may be used to link the rotational speed of cells that are subjected to comparative centrifugal force.

(1) $$\mathrm{RCF}={\left(\frac{\mathrm{RPM}}{1000}\right)}^{2}\times r\times 1.18\;$$

RCF for 200–4000 RPM at the base of the cell varies between 6–2325 g. Only RPM values will be provided in the manuscript’s summary using Equation (1), which makes it simple to convert data to RCF. 

##### Description of Sedimentation Analysis Test

An analytical centrifuge called a LUMiSizer was used to examine phase separation (LUM GmbH, Germany). By subjecting a sample solution to centrifugal velocity higher than that caused by gravity on Earth, the LUMiSizer speeds up deposition. More to 12 samples were investigated at once. Each sample was put into a compartment made of rectangular polyamide (LUMiSizer cell type 3). Each sample was loaded into the LUMiSizer with the top end positioned horizontally along the radial axis, sealed, and closest to the rotational centre. According to calculations, the base of the LUMiSizer sample is 130 mm away from the rotating centre.

For each sample under study, three measurements were conducted again, and the average findings were given. Centrifuge rotation speeds of 200–4000 rpm (at a temperature of 298.15 K) were used in sample analysis at a temperature of 25 °C. A side of every sample cell of the LUMiSizer is subjected to a light source that pulses near-infrared light (865 nm) light at user-specified intervals. The light intensity was generally changed before every run. A 25 mm 2048 element CCD-line is used to measure the amount of light that is transmitted throughout the length of the sample and create transmission profiles. A profile was obtained every ten seconds until the specimen content had completely sedimented (although varied across experiments). The instrument program automatically smoothed each transmission profile using a 9-point moving average.

Volume concentration percent and transmission light intensity are related, according to Lambert–Beer. According to the Lambert–Beer law [42], a high transmission intensity corresponds to a relatively low volume percentage. The intensity of light that is transmitted is inversely proportional to the volume concentration of the particles. Each transmission profile shows a considerable and quick shift in transmission intensity near to the supernatant–suspension interface because the suspensions are essentially monodispersed. The “front tracking” module of the LUMiSizer programme determined the interface height for each profile (SEPView 6.2). The transmission intensity connected to the interface is determined by a transmitting threshold. Because the suspension was essentially monodisperse, it was discovered that the interface elevation only slightly changed according on the transmission level chosen. The interface point was therefore represented by 50% transmission intensity. A data series of interface heights calculated under centrifugal force for every sample is the LUMiSizer’s ultimate output.

##### Centrifugal Force

Changes to the sedimentation model can be made to account for centrifugal force circumstances. As a result, it is feasible to include the results of LUMiSizer experiments directly. Understanding acceleration can help one understand particles in suspended silt as seen in Equation (1). Centrifugal acceleration is given by Equation (2).

(2) $$u_{T}=\frac{d^{2}\left(\rho_{d}-\rho_{c}\right)a}{18\mu_{c}}\;$$

Profiles are subjected to a centrifugal force commensurate with a rotational velocity of, say, 400 rpm inside the LUMiSizer for our study, with the cell employed for analysis, and with a spatially varying spectrum of centrifugal velocity from 18× *g* at the meniscus to 23× *g* at the bottom (assuming a 27 mm sample height). The bottom and meniscus accelerate at centrifugal forces that are 27.7% different from each other.

##### Earth Gravity Conversion 

The result of the LUMisizer is a time-series data of contact heights that were measured under centrifugal force. In order to determine the detachment velocity, v(t), which is generally the variable of interest, each time series must be translated into Earth gravity conditions. When using centrifugation in suspension stability tests, it might be challenging to accurately convert the data collected under centrifugal force to Earth gravity circumstances. 

Many of these transitions have been published in the literature. Lerche and Sobisch [43] provide a method using the initial separation velocity and the relative centrifugal force (RCF) at the meniscus, $x_{max}$. The RCF is the proportion of gravitational acceleration to centrifugal acceleration. Their method has a serious flaw in that the noise-prone nature of light transmission profiles near to the interface makes it difficult to determine the initial separation velocity properly. Tehrani-Bagha [44] also showed that extrapolating results from experiments utilizing numerous RCF values does not adequately recover the separation velocity at Earth gravity. Here, it is proposed to convert the interface’s spatially averaged RCF to Earth gravity settings. Taking into account an interface height $h_{c}$ that was obtained at time, $\;t_{c}$, while being spun by a centrifugal force, the spatially averaged RCF is calculated by averaging the RCF at the meniscus and the RCF at the radial point corresponding to the interface.

(3) $$\tau =\frac{{\left(2\pi n\right)}^{2}}{2g}\left(\left(r_{max}-h_{c}\right)+\left(r_{max}-x_{max}\right)\right)\;$$

For n (number of centrifuge revolutions per second) given, one term comes from the meniscus and the other from the interface. Because centrifugal acceleration depends linearly on *r*, an interface height $h_{c}$ (attained at time $\;t_{c}$ under centrifugal force conditions) is reached at time $t_{e}$.

(4) $$t_{e}=\tau \;t_{c}\;$$

The recommended conversion makes sure that the impact of suspension concentration and interface location on time, $\;t_{c}$, is considered. For each interface height obtained under centrifugal force, the relevant time may be converted into an identical time in gravity using Equation (4). By conducting this transformation for every point of data given, collected data under centrifugal force can be used to recover a times series of interface depths in Earth gravity settings.

Knowing about the accelerated sedimentation in this case can help with the study of sedimentation when sediments are merely subjected to gravity. The subject of conversion to earth gravity condition is crucial because it may be used to estimate sediment thickness and the yield stress that goes along with it in massive clarifiers used in wastewater and tailings management facilities. If the raw data presented here must be translated to the length of time the settling rate would take if samples were just subjected to gravity forces, Equations (3) and (4) can be employed. 

##### Determining the Effective Maximum Volume Fraction

Maximum packing of particles is another colloidal property that needs attention after sedimentation; accurate representation of maximum packing is crucial, for instance, for land reclamation tailing ponds regeneration in oil sand processing [45]. Moreover, the need for land expansion through land reclamation arises from the strong demand for land in coastal cities or areas nearby. Unfortunately, the compressible estuary or marine deposits that make up the foundation soils of coastal communities are frequently recent deposits. As a result of the complex reclamation and soil enhancement procedures required, reclamation at the foreshore area becomes more difficult. With time, regions with good soil conditions are depleted, and land restoration is even necessary on newly dumped ultra-soft soil and waste ponds.

Further, the flocs formed following flocculation with polymer respond differently during load application than a typical kaolinite would under traditional one-dimensional consolidation; the softer flocs produced suffer a significant amount of deformation at first with little to no effective stress gain. Due to the highly soft nature of the reclaimed ground, special techniques are needed and, in certain circumstances, additional chemicals are needed to reinforce the foundation soil. It is more difficult to forecast settlement with soft flocs than with bare kaolinite. The magnitude for achieving the same compressive strength is frequently underestimated since the right theories are not being used. It is therefore vital to undertake research on maximal packing and how it differs from PAM flocculated kaolin. 

Maximum packing can very easily be estimated using sediment bed thickness obtained through LUMisizer. Maximum packing, based on wide spread literature results and theories, is also under electrostatic charges; in fact, the effective maximum volume fraction, $\varphi_{max}^{eff}$, is given by [46]:

(5) $$\varphi_{max}^{eff}=\varphi_{max}{\left(\frac{d+p}{d}\right)}^{-3}\;$$

where *p* is the minimum separation distance between the surfaces of two neighboring articles and $\varphi_{max}$ is the low shear maximum volume percentage of randomly packed hard spheres, with the value 0.639 adopted here as determined by the authors in ref. [47]. It is noteworthy that, under pressure, the sediment bed is compressed toward its maximum volume percent; however, this volume fraction is unlikely to fully materialize. This equation will be used later as the sedimentation data are assessed simultaneously.

## 3. Results and Discussion

### 3.1. Dynamic Light Scattering

To ascertain the effects of adding PAM to kaolinite at a concentration of 0.1 weight percent on particle sizes, samples were examined using dynamic light scattering. In order to get the samples ready for analysis, PAM was added to the mixture at doses of 7.5 and 15 ppm. One minute after sedimentation, samples were taken from the super-natant suspension after a brief period of stirring. The samples looked to have been overdosed and high amounts of kaolinite particles remained in the supernatant.

**Figure 2:**
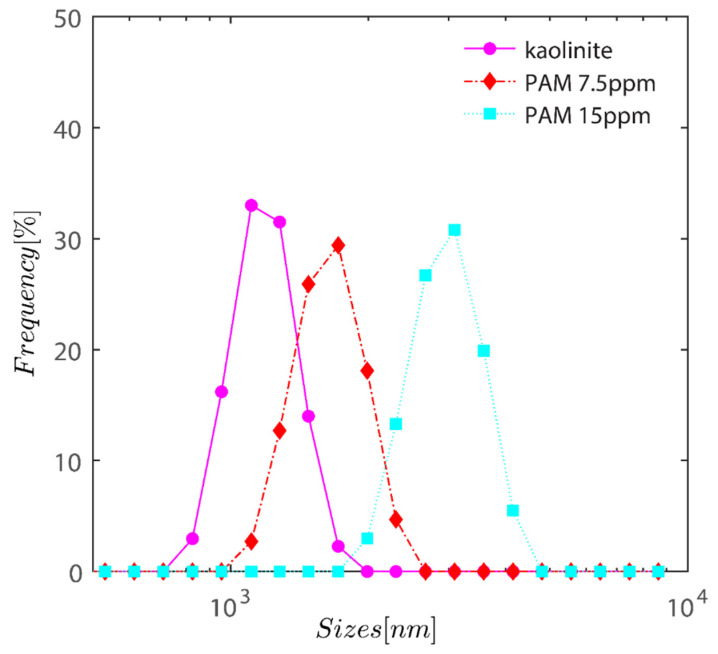
Semi-logarithmic variation of volume percentage as a function of hydrodynamic radius of kaolinite. The graph displays a gradual shift in volume-averaged particle size as PAM dosage increases from 0 to 15 ppm.

Figure 2 demonstrates the semi-logarithmic change in volume % as a function of kaolinite hydrodynamic radius. The graph shows an increase in PAM dose from 0 to 15 ppm as a steady change in volume-averaged particle size. With the addition of PAM, the volume average size of the particles has changed from 1 µm for bare kaolinite to 1.7 µm for PAM flocculated flocs at a dosage of 7.5 ppm, and eventually approaches 3 m when the PAM dosage is increased to 15 ppm as expected owing to the particle being bridged with PAM.

### 3.2. Transmission Electron Microscopy

**Figure 3:**
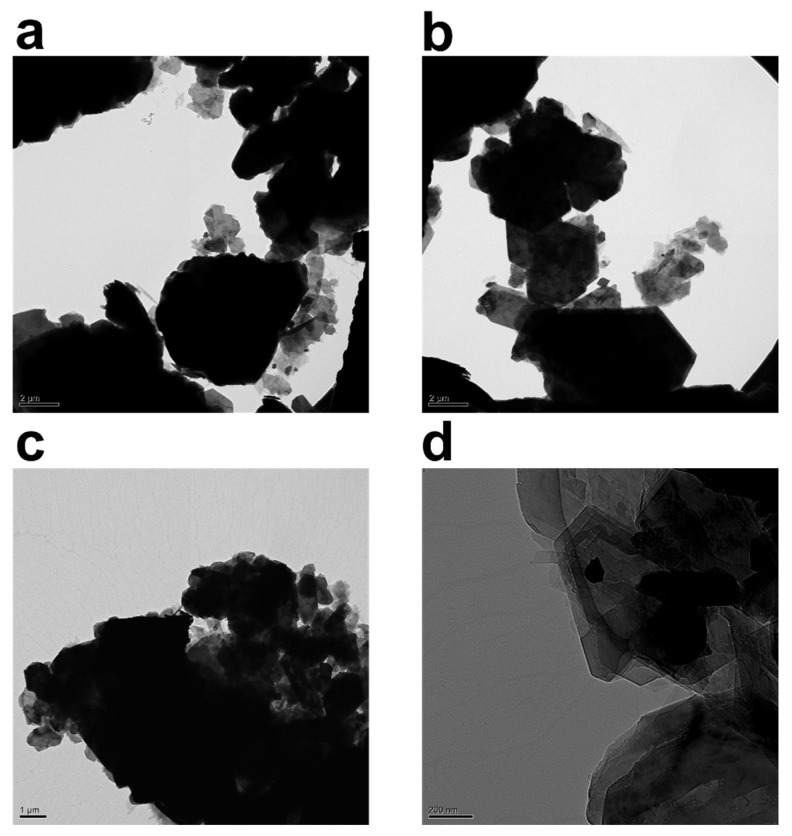
TEM images of 2.5 wt% kaolinite suspension placed on a carbon grid: (**a**,**b**) without non-anionic polyacrylamide (PAM), and (**c**) with 0.08 wt% (800 ppm) PAM and (**d**) 0.1 wt% (1000 ppm) PAM. Content of PAM and magnification are tuned for best visibility.

The TEM images of kaolinite particles in the water suspension in the absence and presence of PAM are depicted in Figure 3. Figure 3a–d reveals the triclinic hexahedral shapes and hexahedral structures famously associated with kaolinite particles. As previously reported in the literature [10], in suspensions without polymer, the kaolinite particle associations have a bulky and spider-web-like structure. Figure 3a,b depicts bare kaolinite particles, whereas Figure 3c,d depicts samples that have been flocculated with 800 ppm and 1000 ppm PAM, respectively. With the inclusion of PAM, the sample has clearly clustered. 

Figure 3 collectively attempts to depict morphological changes monitored through transmission electron microscopy (TEM) after the addition of the polymer flocculant into the system. The black hexagonal features attached to the PAM chains (network) are kaolinite particles. The crucial value for PAM’s energy per segment must be exceeded in order for polymer chains to adhere to the surfaces of the particles, and this threshold is demonstrated in ref. [48]. Only then can PAM be connected to the particles. Since polymer chains lose their freedom of conformation and translation during attachment, the energy loss must be made up for by the energy of adsorption. The loss-in-translation term is minimal for longer polymer chains because loss of conformational flexibility dominates the overall entropy loss term.

Description of attached polymer chains is commonly made through trains, loops, and tails. The TEM images captured in Figure 3 clearly demonstrate that un-adsorbed polymer chains contribute to the thickening of the polymer structure around kaolinite particles. Moreover, PAM chains seem to be quite effective at holding large clusters of kaolinite particles from beneath. In Figure 3c,d, darker aggregates are kaolinite clusters, while grayer aggregates are a polymer layer that connects the particles together; it is possible to statistically track sizes of clusters with TEM, and fractal dimension can also be estimated; however, due to a lack of knowledge on spatial positioning and number of kaolinite particles in each aggregate, as well as sample preparation tampering that can influence particle size, we have refrained from providing this statistic. 

Variation in the morphology of the polymer chains surrounding kaolinite particles reveals two prominent features. First, when the concentration of the polymer in the system is low, the polymer structure is branchy, while, at higher concentrations, the polymer structure acts as a sheet covering kaolinite particles by holding the particles together. Lee et al. [10] visualized quite similar morphology in a CaCl_2_-PAM system. An increase in branch length happens through association of hydrophobic groups on polymer chains, leading to the enlargement of polymeric entities. It is noteworthy that non-anionic PAM has tendency, seemingly, to adsorb to both sides of the kaolinite particle as reported elsewhere [8,49,50]; therefore, the PAM investigated here may specifically adhere to and flocculate both silica and alumina particles (a constituent of kaolinite with alumina making roughly 30% of kaolinite chemical makeup); nevertheless, the effectiveness of each PAM must be determined separately. 

The TEM pictures displayed here can serve as an example of how clay particles might be arranged given their propensity to agglomerate to the basal plane. We attempt to estimate maximum packing, which is a collection of particles constructed layered on top of one another, later in the maximum packing section.

### 3.3. Scanning Electron Microscopy of Freeze-Dried Flocs

**Figure 4:**
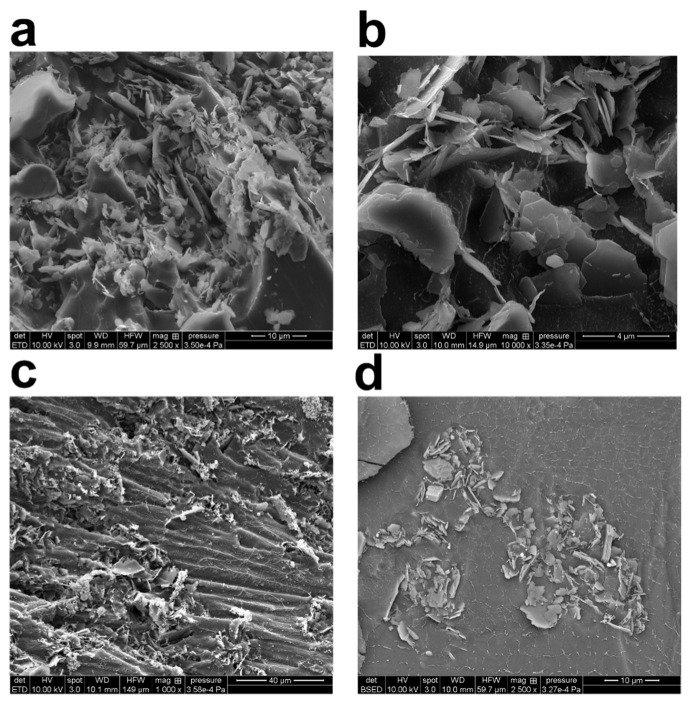
High magnification cryo-scanning electron microscopy (SEM) images of freeze-dried 2.5 wt% kaolinite/water suspension: (**a**) with 0.08 wt% (800 ppm) PAM flocs; (**b**) with 0.1 wt% (1000 ppm) PAM flocs; (**c**,**d**) with 0.3 wt% (3000 ppm) PAM flocs. Content of PAM and magnification are tuned for best visibility.

To investigate the structure of the generated flocs, the kaolinite suspensions made at three different polymer concentrations were freeze-dried and imaged with the SEM setup (Figure 4). Figure 4a,b shows samples of freeze-dried raw clay, whereas Figure 4c,d shows samples that have been grouped together with PAM. The flocculated clay slurries were prepared according to the process set out in Section 2.2 and used for the cryo-SEM testing. SEM image analysis reveals that the kaolinite particles subjected to forced sonication have been evenly distributed all through the sample. Additionally, bridging flocs’ architecture may be seen by cryo-SEM imaging. The principal kaolinite nanoparticles found in the micro-flocs range in size from 20 nm to 100 nm. The micro-flocs were nearly continuous or had long polymer chains separating them from one another.

Cryo-SEM was also used to examine the principal particle orientation and organization inside the kaolinite micro-flocs. There were several edge-to-face and face-to-face interactions visible throughout the field of vision (Figure 4). The close-ended polymer chains create web-like structures that connect different micro-flocs to each other (in agreement with TEM images). It was possible to see that the sizes of these polymer chains varied. Based on the study of TEM and SEM micrographs, the PAM chains’ thickness ranges from a few nanometers to roughly 80 nanometers. The clustering of thin polymer chains, which results in the formation of thicker chains, or the existence of undissolved polymer, may be the causes of the variability in polymer chain dimension. In their hydrated condition, the polyacrylamide chains have both positive and negative sites. There is a possibility of aggregation owing to electrostatic forces, depending on the relative alignment of these units in the polymer chain. These possibilities are based on visualizations that were made to show the water loss from cryo-freezing and sublimation, which may have caused the thin polymer network to collapse and resulted in bulkier polymer chains.

Given that the shape of the flocs produced at the concentration under study is quite loose, the results of the SEM and TEM sections may be related to the subsequent section. The quality of the flocs’ looseness facilitates understanding of the sedimentation speeds that LUMisizer would later produce. It is interesting that in both TEM and SEM pictures, the sizes of the particles are in micrometer range. The size of the particle later will also be examined with DLS. 

### 3.4. Settling Behavior Analysis

**Figure 5:**
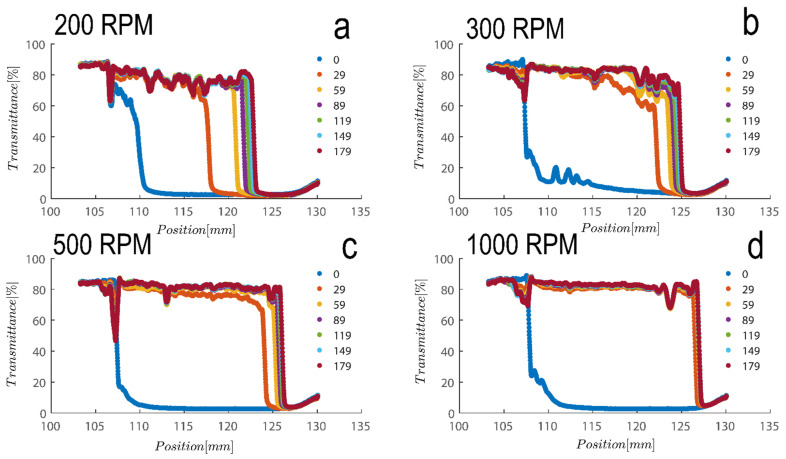
Transmission profiles obtained during centrifugation of a kaolinite sample (Sigma I) with 7wt% at (**a**) 200, (**b**) 300, (**c**) 500 and (**d**) 1000 rpm (corresponding to a centrifugal acceleration of 5.1, 11.3, 31.5, and 126× *g*, based on middle point of 115 mm). Volume of suspension added into the cells is 0.4 mL. Position of illuminated cell varies between 103–130 nm; the low transmission at the end of the cell demonstrates that particles are accumulating there.

The settling velocity of the particles under various gravitational forces may be used to assess the stability of the separations [51]. The stability of kaolinite suspensions at various RCF (relative centrifugal force) levels is depicted in Figure 5 for Sigma I sample. As anticipated, raising the RCF enhanced the samples’ settling velocity [52]. The figure’s legend denotes the experiment’s timing, which runs from 0 (i.e., the beginning state of suspension) to the final position of suspension at 179 s of centrifugation. There is frequently a linear correlation between settling velocity and RCF [43,53], which lends credence to the idea that the particles are monodisperse and that Stokes’ equation may be utilized to analyze the samples. Overall, it appears that the shape and porosity of the particles may have a significant impact on the settling velocity of the particles [43,53], as will be demonstrated.

Figure 5 shows a transmission profile that was obtained by centrifuging Sigma I at a concentration of 2.5 vol%. The profiles were subjected to a centrifugal force sustained with a rotational speed of, say, 4000 rpm in the LUMiSizer. The range of centrifugal velocity varied spatially, ranging from 1807 g at the meniscus to 2280 g at the bottom (a 27 mm sample height). Figure 5a–d shows samples centrifuged at 200–1000 RPM as indicated on top of each figure. Therefore, there is a 26.2% discrepancy in the centrifugal force between base and meniscus. Using the previously discussed Equations (1) and (2), the size of the particles based on the speed of the interface separation in Figure 5 can be estimated; at 200 rpm, the average separation velocity of the particle can be estimated to be 63 µm/s over a period of 200 s for the Riedel sample at a concentration of 7 wt%; roughly speaking, the size of the particles can be estimated to be 4 micrometers using combinatory Equations (1) and (2), which is consistent with our previous calculations using dynamic light scattering. 

Additionally, as samples are shear thinning, they are expected to become oriented towards flow direction; here along in longitudinal direction of cells towards the sediment bed, this rate of migration is expected to be expedited due to the higher rotation pace of samples in LUMisize. The predicted separation velocities of the particles at 300, 500, 1000, and 4000 rpm are therefore estimated as 90, 95, 100, and 352 µm/s, respectively. It is approximated here using an average point of 50% transmission from time=0 until the time profile reaches the sediment bed, albeit the technique of assessing sedimentation can vary. We will later demonstrate a different approach to estimating sedimentation speed that is based on the examination of transmission raw data at a height of 130mm. Moreover, as particles become separated and accumulate close to sediment bed, separation velocity becomes hindered as Equation (1) is no longer applicable, and the movement of particles is hindered due to neighboring particles. The hindered settling velocity is given by

(6) $$u\left(\varphi \right)\approx uf_{h}$$

where $f_{h}$ is a function based on volume fraction and is calculable based on the phenomenological Richardson–Zaki relationship [54] or Acrivos’ hindered settling function [55]; that is, the settling rate based on equation 1 is slowed down when concentration is increased and passes a threshold. Discussing the effect of hindered velocity due to crowding with surrounding particles is out of scope of current manuscript. Moreover, the settling rate speed calculated earlier can be superimposed to a situation in which centrifugation acceleration is substituted by gravitational acceleration; in this way, sedimentation of suspension over time can be estimated (see Equations (3) and (4)). These estimations are important for prediction of stability of clay suspensions over the long term.

As an alternative, sedimentation speed may also be calculated by measuring the growth of the sedimentation bed over time and calculating a derivative of the increase in thickness over time. The approach was explained in detail in ref. [56]. 

**Figure 6:**
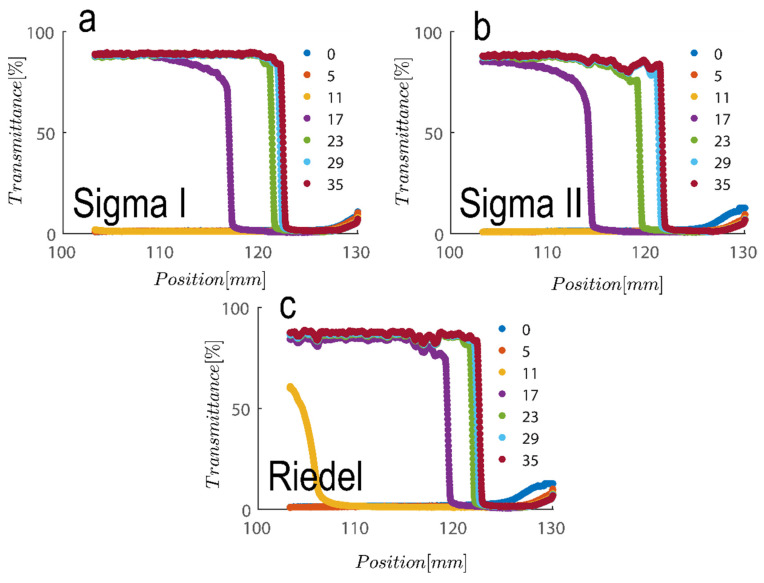
Transmission profiles obtained during centrifugation of a kaolinite sample: (**a**) Sigma I, (**b**) Sigma II and (**c**) Riedel at 14 wt% at 4000 rpm for duration of roughly 30 s (corresponding to a centrifugal acceleration 2017 g, based on middle point of 115 mm). Volume of suspension added into the cells is 1.1 mL. Position of illuminated cell varies between 103–130 nm; the low transmission at the end of the cell demonstrates that particles are accumulating there.

Another colloidal property severely involved with particle size and geometry is maximum packing volume fraction. This is based on the experiment performed in Figure 6 for three samples examined are compared; to achieve this, first, the cross section of the cell was calculated by adding 1.1 mL of water filling out the thin portion of cells and subsequently measuring the cell height at 50 mm, which leads to a calculation of the cross section at roughly 22 mm^2^. Maximum packing is therefore calculable upon the addition of suspension and measurement of the sediment bed at the kaolinite concentrations examined here. The sediment bed is considered the point at which transmittance sharply decreased based on the results in Figure 6. Table 1 contains a summary of the analysis. As can be shown, the maximum packing for 2.7 vol% (7 wt%) and 5.4 vol% (14 wt%) suspensions for Sigma I, Sigma II, and Riedel sample, respectively, is about 40 and 42, 27 and 33, and 34 and 38. With maximum packing, one can produce a reliable estimate of the aspect ratio of the particles. The rod glass regime is bounded on the top by the maximum geometric packing fraction of isotropic rods [57].

(7) $$\varphi_{max}\approx \frac{5.4}{r}$$

In which r is the aspect ratio of particles—here, length over thickness of hexagonal kaolinite particles—and $\varphi_{max}$ is maximum packing. Based on maximum packing values obtained here, the aspect ratio of particles is varied roughly between ~12–20 across the three samples examined; these values are in line with values reported in our previous publication using confocal laser scanning microscopy [30]. 

### 3.5. Sedimentation Behavior in Presence of PAM

**Figure 7:**
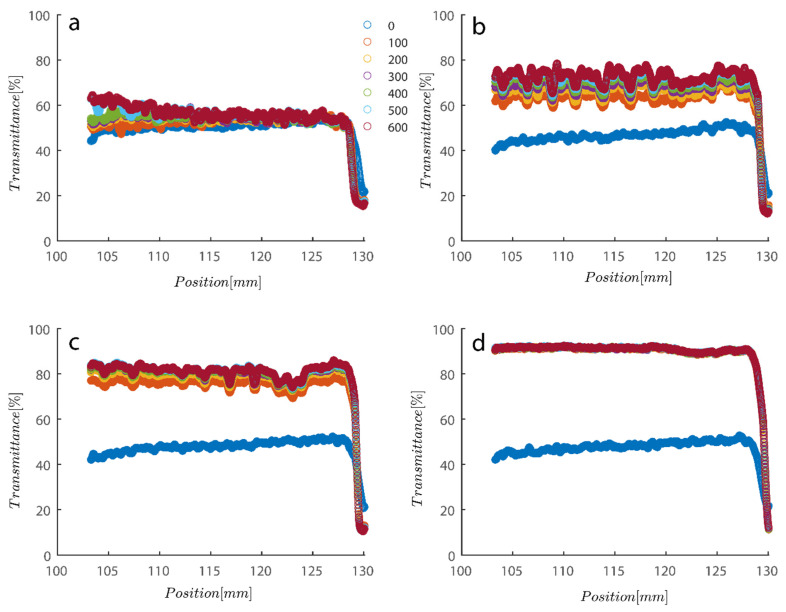
Transmission profiles obtained during centrifugation of a kaolinite sample Sigma I: (**a**) 200, (**b**) 500, (**c**) 1000, (**d**) 4000 RPM at 0.1 wt% concentration. Position of illuminated cell varies between 103–130 nm; the low transmission at the end of the cell demonstrates that particles are accumulating there.

As previously mentioned, softer flocs with additional PAM may act differently during centrifugation; consequently, this section’s agenda includes an analysis of each type of behavior. Figure 7 depicts the initial settling rate behavior of a kaolinite sample subjected to centrifugal rotational speeds of 200× *g*, 500× *g*, 1000× *g*, and 4000× *g* RPMs to provide a baseline for comparison. It is obvious that as rotational speed has increased, the amount of time needed to improve transmission has decreased from the initial transmittance of around 50% to 80% and 90% as centrifugation was completed in 600 s (or 10 min). 

**Figure 8:**
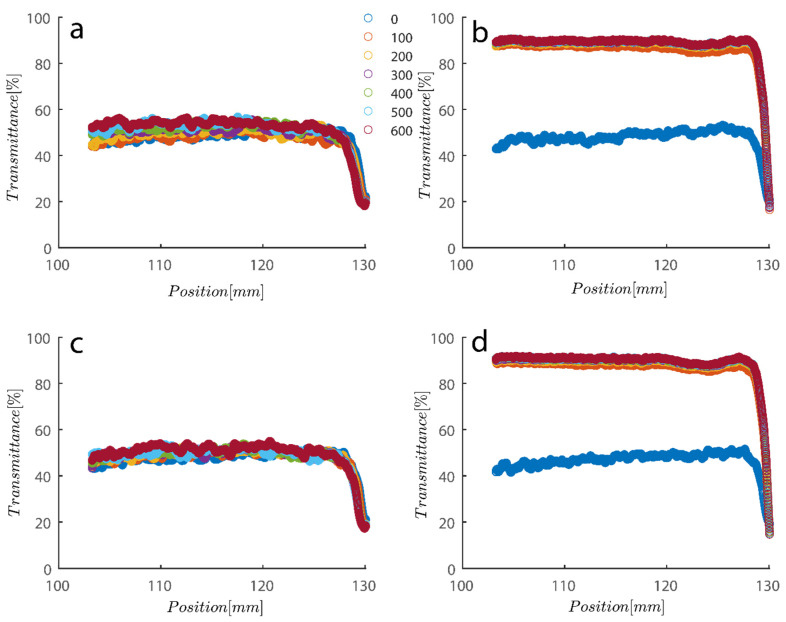
Transmission profiles obtained during centrifugation of a kaolinite sample Sigma I (**a**) 200, (**b**) 4000, (**c**) 200, (**d**) 4000 RPM. At 0.1 wt% concentration with (**a**–**c**) 7.5 ppm PAM and (**b**–**d**) 15 ppm PAM added into the mix. The position of the illuminated cell varies between 103–130 nm; the low transmission at the end of the cells demonstrates that particles are accumulating there.

As previously mentioned, 7.5 and 15 ppm of PAM were added to the cells, and samples were exposed to centrifugal forces, this time utilizing the rotating speeds of 200 and 4000 RPMs for both cases, to evaluate the settling behavior of un-flocculated and flocculated kaolinite samples. It is obvious that sedimentation brought on by a potential PAM overdose on the system has rendered settling slower; however, more study of the raw data in Figure 7 and Figure 8 is required to quantify this feature. It is noteworthy that while transmission of bare kaolinite increased from 40% to 90% for most of the cell length in less than 100 s, the same development for samples overdosed with 7.5 and 15 ppm rendered floc separation from the solution more difficult. 

**Figure 9:**
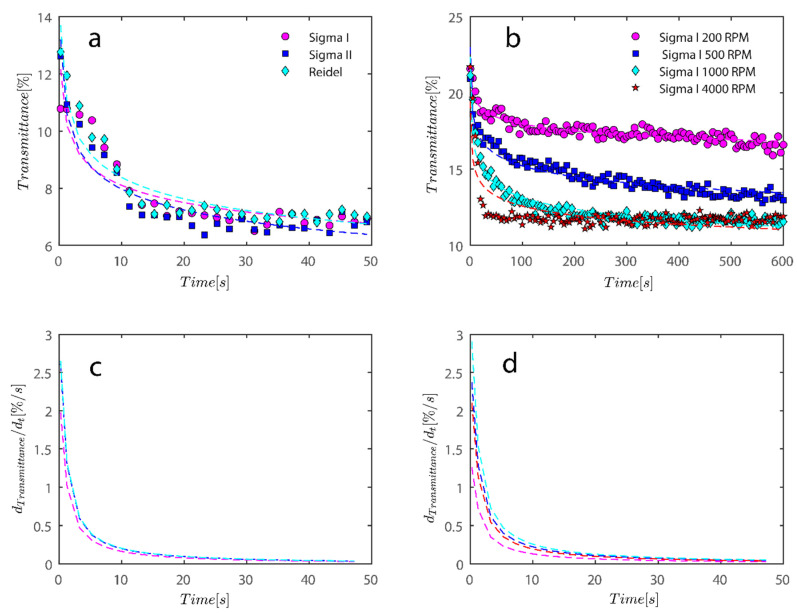
For the data shown in Figure 5 and Figure 6, an analysis of the sediment bed at a height of 130 mm as a function of time was performed. (**a**) The reduction in transmission intensity for the Riedel, Sigma I, and Sigma II samples at the cell’s roughly 130 mm end as a function of time at rotational speeds of 200, 500, 1000, and 4000 rpms; (**b**) reduction in transmission intensity for Sigma I samples with time at rotational speeds of 200, 500, 1000, and 4000 rpms at a point 130 mm or so from the cell’s end; (**c**) the power law’s derivative fits in Figure 8a, and related information is shown in Table 2. The data that correspond to (**d**) derivative of the power law are shown in Table 2 along with Figure 8b.

To quantify the behavior seen in Figure 7 and Figure 8, a simple power law fitting was applied to the data, and the results are shown in Figure 9 to provide a rough estimate of the rate of sedimentation as a function of time. Rates of decay of transmission profiles at position 130 (end of analyzing cell) were compared as a function of time for different RPMs for data shown in Figure 6 and Figure 7. For all three of the samples that were previously analyzed, measuring data in Figure 9a shows that sample transmission has steadily decreased under 4000 RPM centrifugal forces. Sigma I, Sigma II, and Riedel samples clearly show that differences in electrostatic forces and shape have minimal bearing on the way that kaolinite samples segregate. Another peculiar observation is that the sediment bed reached a steady state as approximately 20 s passed. Sigma I sample under centrifugal forces also shows interesting behavior as centrifugal forces’ magnitude was heightened from 200 to 4000 RPMs. It is evident from the data that samples exposed to stronger centrifugal forces reached a steady state more quickly and maintained it for the duration of the experiment; samples exposed to weaker centrifugal forces, however, took longer and the sediment bed appeared to permit more light to pass through; consequently, the steady state condition in these cases is far from maximum packing and the sediment is still very loose even after 600 s have passed. 

Additionally, using finite difference numerical methods or using the derivative of the fitted power law equation in Figure 9a,b, the rate of transmission degradation over time may be calculated with ease. Initially, the rate of decay is high as shown in Figure 9c,d; however, in a short amount of time, it approaches zero as sediment compressive yield stress balances out the centrifugal forces; discussion on compressive yield stress is postponed to later sections. One may compare the average later rate of degradation during the investigation to the sample flocculated with PAM. 

It is intriguing that when the rotating speed increases to 4000 RPM, data no longer fits the equation as well and appears to be departing from the power law fit. This may be because the sedimentation process has changed the mechanism from what was observed before at lower RPMs. To address these variations, more research is required. 

**Figure 10:**
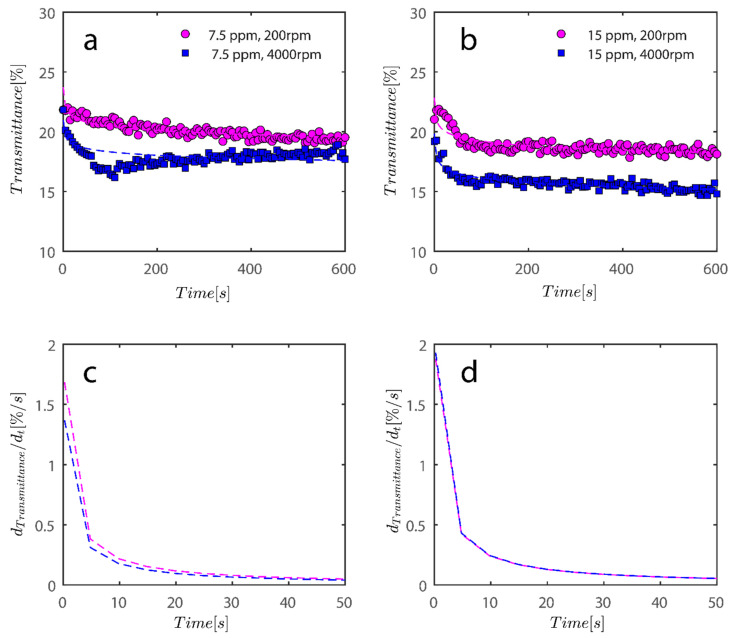
For the data shown in Figure 8, an analysis of the sediment bed at a height of 130 mm as a function of time was performed. (**a**) The reduction in transmission intensity for the Sigma I flocculated with 7.5 ppm PAM samples at the cell’s roughly 130 mm end as a function of time at rotational speeds of 200 and 4000 rpms; (**b**) reduction in transmission intensity for Sigma I samples with time at rotational speeds of 200 and 4000 rpms at a point 130 mm or so from the cell’s end; (**c**) the power law’s derivative fits in Figure 10a, and related information is shown in Table 2. The data that correspond to (**d**) derivative of power law fit are shown in Table 2 along with Figure 10b.

To compare the analysis done in Figure 9 with flocculated samples, the same analysis was done for samples flocculated with 7.5 and 15 ppm PAM and the results are shown in Figure 10. 

Changes in the sediment bed are not as drastic as naked kaolinite because the addition of PAM has probably led to overdosed particles finding it difficult to accumulate themselves at the bottom of the analyzing cells. The behavior of the rate of decay of transmitted light over time seems to follow the same behavior as naked kaolinite. 

To quantify the explanation and data presented graphically, data has been tabulated in Table 2. Data tabulated are samples, the condition of the experiment, and the mean speed of settling rate obtained from derivatives of power fits. Three kaolinite samples at 4000 rpm centrifugal rotational speed provide a similar mean speed of sedimentation. Sample Sigma I, as rotational speed increases, displays incrementally higher settling rates because the settling rate increases from 0.16 to 0.33 as RPM increases from 200–1000; however, at 4000 mean speed, it shows a decrease, which is due to the inability of the power law to fit the data well as RPM increases to 4000. Looking at data in Figure 9b qualitatively reveals that the mean speed on average is higher for 4000 as opposed to 1000. It is clear from the mean settling rates values listed in Table 2 that the compaction of the sediment bed has slowed by over an order of magnitude with the addition of PAM in quantities of 7.5 and 15 ppm. Flocs with an uneven structure can exhibit greater resistance to centrifugal forces. 

Through the study of transmission data from the top layer and the mean volume fraction of particles based on the Lambert–Beer law, the hydrodynamic diameter of the suspended particles in the system may be ascertained:

(8) $$T\left(l,r_{i}\right)=T_{0}e^{-\frac{2r_{i}}{l}}\;$$

(9) $$l\left(d_{h,s},\varphi_{s}\right)=\frac{2d_{h,s}}{3\varphi_{s}Q_{s}}\;$$

In these equations, $r_{i}$ is a representation of the internal radius of the measurement cells, l is the mean free path of photons, $T_{0}$ is the transmittance associated with the continuous phase (here water) and T is the transmittance of suspension (i.e., kaolin suspension) [51,58]. As a result, the instrument’s collection of transmission data is directly influenced by the particles’ mean hydrodynamic diameter, $d_{h,s}$, and their particle sizes, $\varphi_{s}$. 

Results of the LUMisizer analysis in terms of median and harmonic mean sizes of particles is shown in Table 3; data is tabulated as a condition of the experiment, kaolinite concentration, and PAM concentration. As the centrifugal force increased, however, the initial clusters broke down to smaller cluster sizes that at 4000 RPM reaches below micrometer size. The size of the particle estimated by LUMisizer software is initially bigger as at 200 RPM, the size of the particle’s hovers around 10 µm. Similar trends can be seen with PAM flocculated samples. 

The maximum packing was also investigated as a function of the amount of PAM added. Experiments were conducted by stirring a jar tester at 250 rpm in a 600-mL beaker with a 400-mL total solution volume. Later, samples were centrifuged at 4000× *g* rpm for 10 min. Maximum packing decreased at 7 wt% kaolinite from 46 ± 2 to 35.6 ± 0.1, 35.6 ± 0.2, 34.0 ± 0.2, and 33.7 ± 0.1 vol% as the PAM concentration changed from 0, 3, 6, 15 ppm. PAM caused flocs to expand, which lowered the maximum packing when PAM was present. 

### 3.6. Simulation: Lubachevsky-Stillinger (LS)

In the previous section, maximum packing was experimentally analyzed; here, simulations will be used to simulate the maximum packing. Three simulations will be shown in detail and examined here; the effects of the simulations on random tight packing, the compression rate of the computation processing, and the change in polydispersity will be explored. The findings of the simulations will be compared with the experimental values discovered earlier in the next section. 

Generally, physical compression methods in the form of simulations and experiments frequently involve a hard container boundary that is compressing, such as a piston pressing on the particles. The Lubachevsky–Stillinger (LS) algorithm can be used to model such an event [59]. The LS was initially introduced in a situation without a hard boundary, while the virtual particles were “swelling” or developing in a fixed, limited virtual volume with periodic boundary restrictions [60,61]. As the particle absolute sizes grew, the relative sizes between the particles remained constant. The LS algorithm can theoretically handle simultaneous external compression, internal particle expansion, a potential but optional hard barrier, and more. It is also possible to make the border mobile. 

Some particles can move inside “cages” made by their immobile, jammed neighbors and the hard boundary, if existent, rather than being stuck, is in the final, compressed, or “jammed” condition. These free-to-move particles are not an artifact, a pre-designed property, or a goal feature of the LS; they are a real phenomenon. The simulation revealed this tendency, which surprised the LS authors. According to Frank H. Stillinger, the free-moving particles are called “rattlers” because they will ratchet if a compacted collection of hard particles is physically shaken. 

The compression and expansion can be halted if requested in the “pre-jammed” mode whenever the arrangement density is low, and the particles are mobile. The LS would simulate a granular flow in this scenario. It is possible to represent different instantaneous impact dynamics, like those with and without tangential friction and complete restitution. It is also possible to consider variations in particle masses. Reducing all or some of the particles makes it simple and sometimes beneficial to “fluidize” a congested structure.

Another way to extend the LS is to substitute a piece-wise constant force potential for the hard contact force potential (zero outside the particle, infinite at or inside). The LS would roughly replicate molecular dynamics by continuously interacting with short-range particles. If it is possible to quantify the inter-collision velocity of each particle using a straightforward one-step computation, and external force fields such as gravity can also be considered. The use of the LS for spherical particles of different sizes and/or for jamming in a non-commensurable size container [62,63] was a useful technique for producing and analyzing microstructures produced under the impact of a crystallographic defect or a geometrical frustration. It should be remembered that spheres of different sizes were the early LS protocol’s main target group [64]. 

The equivalent of isostaticity and jamming have been seen in tests, and it has been proven that isostaticity is the required condition ensuring infinite pressure and jamming. Simulations operate on the premise that an ensemble of frictionless particles exhibits collective jamming. Rattling particles are not eliminated from the simulation. In this work, when we refer to jamming, we mean collective jamming in packings of frictionless particles, comparable to mechanical stability, and infinite pressure in particle systems with velocity input. Experimental evidence supports the equality of isostaticity and jamming, whereas literature established that isostaticity is a prerequisite for infinite pressure and jamming [65]. If there is at least one subset of jammed particles in a packing, it is said to be jammed (other particles are rattlers). When calculating packing densities, rattler particles are not excluded from the packings [65,66,67]. 

Other algorithms used in the manuscript to assess the level of maximum packing in the suspensions are the force-biased algorithm (FB) and Lubachevsky–Stillinger with gradual densification (LSGD) [67]. For the FB algorithm please refer to refs. [24,68]. The LSGD algorithm is the modified LS procedure as after 20 collisions each particle with compression packing will become equilibrated. The equilibration is carried out by performing sets of 20 collisions for each particle with zero compression rate in a loop until the relative difference of reduced pressure becomes less than 1% so the pressure becomes steady [28]. 

To explain the FB algorithm very briefly, it is a method that creates irregular tight packing from a random distribution of points. This technique is also known as the “neighbor separation algorithm” (NS) [69,70]. The key advantage of this method is that the diameters of the spheres can change from one step to the next depending on how the ensemble is currently arranged. Each sphere has a different inner and outer diameter. After each cycle, the inner diameter—which defines the actual packing density—is set to the shortest length of two spheres’ centres. Due to the relatively large initial value of the outside diameter, the packing fraction is 1. The outer spheres cross each other as opposed to the inner spheres. This eliminates the worst overlap between both the outer spheres in each cycle by isolating the spheres until their distance reaches the outside diameter.

The simulation was found using the following parameters: 100 particles, a starting volume fraction of 0.58 thus variable box length, a particle diameter set at 1, and the ability to vary the compression rate and polydispersity as shown below. Computer simulations have indicated that a hard sphere can achieve a high-density state above a volume fraction of 0.58 [71] and transition to a random close-packed solid at 0.63. For the demonstration of the effect of polydispersity on packing, the population of particles was assumed to follow a log-normal distribution, and polydispersity was permitted to fluctuate as a stand deviation by Table 4 and Figure 8. Table 4 displays tabulated data as a function of polydispersity and compression rate. Simulation results are summarized in each instance with a visual representation. Details of the simulation and algorithms used can be found in the supplementary section of reference [72]; providing the details the simulation and algorithms is out of the scope of the current study. 

The LS method significantly slows down if ellipsoids (or ellipses in two dimensions) are employed in place of spheres for even the slightest departure from the spherical (or circular in 2 dimensions) [73] form. On today’s standard personal computers, the LS can handle particle configurations in the tens to hundreds and thousands. Nevertheless, there has not been much practice with the LS at dimensions beyond three [74]. Before the simulation began, the polydispersity and algorithmic approach were changed, and the compressing rate was maintained while mostly following ref. [72]’s description and nomenclature.

Because smaller particles occupy the spaces between adjacent big particles, increasing polydispersity is known to boost packing efficiency [64,75,76,77]. This is because smaller particles can pack more tightly. Equations for the packing fraction of polydisperse hard spheres that rely on the characteristics of the particle-size distributions utilized have previously been given [78], as well as for some upper and lower bounds for their RCP fractions. Given that the RCP percentage is dependent on the process used to create the packings [78], the greatest packing fraction of polydisperse hard spheres that can be achieved is still unknown.

**Figure 11:**
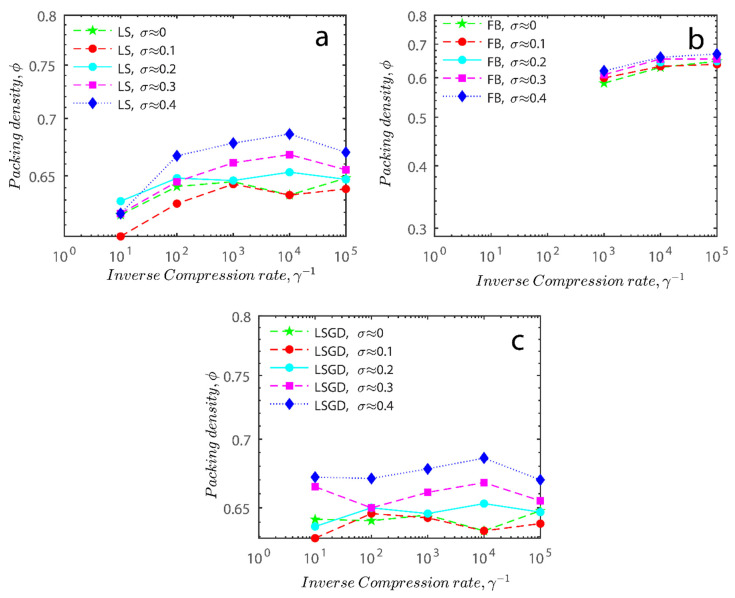
Inverse compression rate vs packing density (volume fraction) estimated as a function of log-normal polydispersity and using three different algorithms. (**a**) The Lubachevsky–Stillinger (LS) algorithm-generated sphere packing densities; (**b**) the force-biased (FB) algorithm-generated densities of spherical packings; (**c**) the LSGD algorithm-generated densities of spherical packings for polydisperse particles.

Figure 11a–c respectively demonstrates packing density variation as the LS, FB, and LSGD algorithms are implemented; results depend on inverse compression rates. The data in the Figure were not averaged; each point in these graphs represents a separate packing generated by the simulations. Points have been connected by straight lines to direct the eye. The assumption behind averaging is that data fluctuations will vanish at the thermodynamic limit. Since this issue is still open, we will not address it here. Averaging would also eliminate details on the precise limits of jamming intervals for limited packings. 

In Figure 11, we can also distinguish between the slow compressions (high inverse compression rates, lengthy generation periods) and the rapid compressions packing creation regimes (i.e., low inverse compression rates, short generation times). For the FB packings with γ^−1^ > 0.2 × 10^4^ and the LS packings with γ^−1^ > 0.6 × 10^2^, we judge the generation to be sluggish based on time dedicated to the simulation. We consider the generation as fast for the FB packings with γ^−1^ < 10^3^ and for LS packings with γ^−1^ < 10^5^. The jamming densities predicted in Figure 11 for all the packing methods stay rather close to the starting densities for slow compressions. 

The packings are essentially crammed and already restricted in closed or nearly closed enclosing zones, which is why this occurs. The hunt for the nearest jammed configuration only slightly increases their densities. The figures for the LS and FB algorithms seem to be similar, despite the fact that the inverse compressing rates for the FB packings are two orders of magnitude higher than those for the LS packings. The following explanation can be used to explain the swift initial compressions seen in Figure 11 as well as the tight horizontal bands for jamming concentrations. Fast cycles make it impossible for the packings to leave (with the Poisson distribution of points as the initial configuration). Just after scanning for the nearest jamming density, packings will still be in their original bounding areas, but the bounding regions will be compressed into polytopes and then jammed configurations, growing slowly the packing density. As a result, the jamming density distribution for rapid compressions should equal the jamming population distribution for Poisson packings, or the uniform sampling of the phase space.

**Figure 12:**
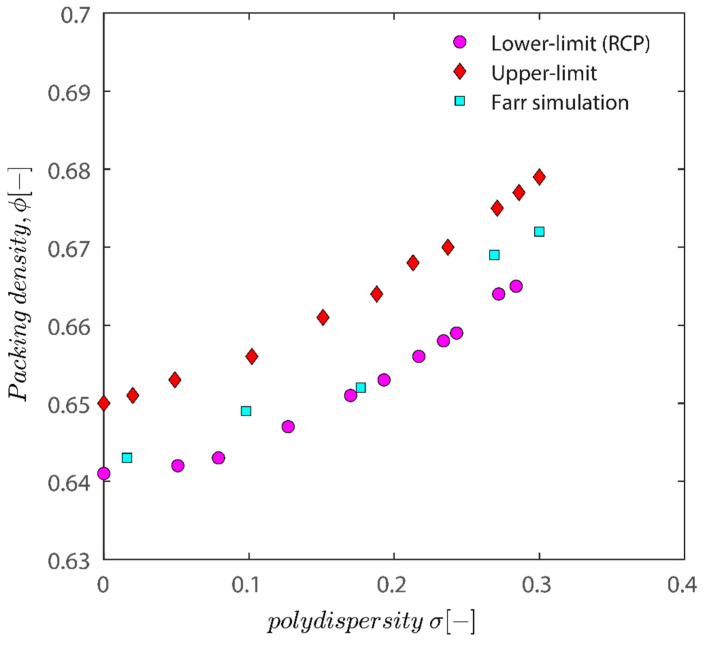
Assessment of the impact of polydispersity on packing density. Comparison between the result of simulation used in this manuscript and results of the semi-theoretical approach of Faar in ref. [79].

Figure 12 demonstrates that all the characteristic densities show an increase with the increase in polydispersity of particles; the increase in the upper limit and the lower limit is natural as polydisperse packing has more degrees of freedom (not limited to location but also freedom with regards to size), and therefore there are more possibilities to arrange them to achieve a certain density. While both the upper and lower limit show an increase in the polydispersity of particles, the difference between the upper and lower limit does not change considerably. The plot of semi-theoretical estimates obtained by Faar [79] also has a similar shape and shifted upwards with an increase in polydispersity, verifying that the results obtained in this study are sound. Ref. [79] simulation results seem to be closer to the lower limit threshold as opposed to the upper limit.

### 3.7. Connection between DLS and Simulation Results

**Figure 13:**
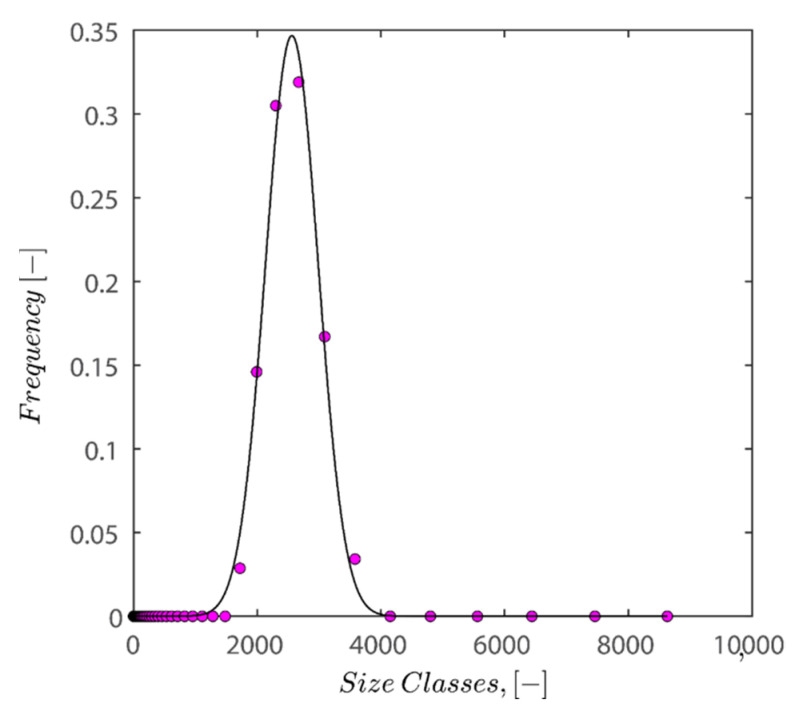
Depiction of the size distribution of kaolin particles under the conditions of pH 7, 25 °C, and 0.1 wt% of kaolin concentration alongside the fitted Gaussian fit distribution.

The Gaussian fit distribution of sizes can roughly describe the volume-averaged distribution shown in Figure 13. The results of fitting the Gaussian fit distribution on normalized sizes are displayed in Figure 13. By dividing particle sizes into average particle sizes, normalization has been carried out, allowing the peak to be located on size classes of 1 micrometer. 

In the next step, 100 random particle sizes are extracted from the distribution with weighted probability explicit in the fitted distribution equation. Then, data was normalized by mean sizes of distribution and fed into the FB, LS, and LSGD algorithms. The result of feeding the particle size distribution depicted in Figure 13 into algorithms leads to random close packings of 0.6219, 0.6219, and 0.6769 for the FB, LS, and LSGD algorithms, respectively. These results are significant as DLS considers particles to be spherical in nature; therefore, utilization of the algorithm is warranted. 

There is a difference between simulation results and the results of experiments as simulation predicts that the maximum packing for spherical particles should occur at 62–67% volume fraction while the experiment showed that the maximum packing hovers around 40–44%. This discrepancy may be caused by several factors, including (i) simulations that incorrectly represent hexagonal geometry as spheres, (ii) insufficient pressure buildup during the application of 4000 RPM centrifugation to achieve random close packing, and (iii) the absence of asymmetrical shifting in particle configurations or vibration during centrifugation, which if active could have caused particle wriggling and higher maximum packings. (iv) A further possibility is that the tight packing of our sample is not random because of the friction between particles, which is crucial for granular packings. Granular packings are frequently looser than random tight packings, as is well known. The simulation’s accuracy in predicting values compared to experimental results is remarkable, nonetheless.

Also keep in mind that the maximum packing percentage for the polydisperse hard-sphere is around 0.75 if their population is log-normal with standard deviation of 0.6 [80]. Desmond and Weeks’ approximation also only applies to close packing densities with standard deviation in the 0 to 0.6 region. The trends show that the maximum random tight packing increases with a standard deviation of the log-normal population, which is in line with the experimental study of refs. [81,82].

The literature needs to pay attention and provide hints on how these equations can be adopted for particles with polymer brushes or particles with adsorbed chemicals such as polymers or surfactants. Some references explored the compaction of plastic particles or soft particles, such as ref. [83]. It is obvious that extending computationally expensive simulations such as the one used here to the case of flocculated samples, though interesting, makes the algorithm very slow and the parameters that need to be considered are not determined. Discussion on the impact of flocculated particles in clusters and maximum packing is out of the scope of the current manuscript.

### 3.8. Compressive Yield Stress Estimation 

The compressive forces (caused by centrifugation) and the strength of the particle network—that is, compressive yield stress at equilibrium—are balanced by the sediment depth. The degree of change in deposit height with rising centrifugal force relies on the stability and topology of the intricate network. Equations may be used to calculate the compressive yield stress when the particle bed has reached equilibrium [84,85]:

(10) $$K_{yield\;stress}\approx \Delta \rho g\varphi_{0}H_{0}\;\left(1-\frac{H_{eq}}{2L}\right)\;$$

where Δρ density difference, g gravitational constant, $\varphi_{0}$ initial volume fraction, $H_{0}$ initial suspension height, $H_{eq}$ is sediment bed height, and L is the length of cell. Plugging in numbers into the equation, assuming initial suspension height to be equal to the length of cell at 50mm, having the height of the sediment bed earlier in addition to knowing RCF at 4000 rpm and considering kaolinite density at 2.6 g/cm^3^ and water at 1 g/cm^3^, leads to the data in Table 5.

Intriguingly, the compressive yield stress of the three materials employed yields comparable findings, indicating that the geometrical variations between the samples did not affect the compressive yield stress of the sediment bed under the conditions of the experiment described in the current publication. In addition to giving useful data, such as the compressive yield stress values in Table 5, analysis of the sedimentation bed and raw data obtained by the LUMisizer are extremely interesting for an estimate of different colloidal characteristics, as shown here. These figures may prove to be quite useful as an additional characterization tool in the bottom-up synthesis of self-assembled soft materials with cutting-edge patterns. When PAM is present, for the maximum packing, which previously varied significantly with its addition, the sediment changes roughly from 3.8 to 4 and the compressive yield stress drops slightly to 48.2 kPa.

## 4. Conclusions

The colloidal behavior of kaolinite during sedimentation in bare form and the presence of polyacrylamide was examined in this manuscript. Various characterization tools, in addition to simulation, were used to evaluate maximum packing, settling rate, and the effect of polymer adsorption on the colloidal behavior of kaolinite particles. According to the results, the maximum packing for the entire kaolinite sample used was about 44 vol%. Sedimentation speed was also discovered to vary based on applied RCF. For the Riedel sample at a concentration of 2.7 vol%, the average particle separation velocity was calculated to be 63 µm/s during a period of 200 s. The separation velocity expedited with the higher rotational speed of the LUMisizer as the predicted separation velocities of the particles at 300, 500, 1000, and 4000 rpm are therefore estimated as 90, 95, 100, and 352 µm/s.

In addition, we discovered that PAM slows down settling by a factor of 10 according to data from LUMisizer. The sediment bed at 4000 rpm reaches a steady state condition around 20 s after the application of centrifugal force starts; this was also observed through the analysis of transmission light data. Due to less centrifugal force being exerted at lower rpm, a steady state took longer to attain. The compressive yield stress for the particles under study was also examined.

First, attempts were made to characterize the charges and sizes of the particles both independently and in combination with a flocculant. The findings indicated that samples display wide polydispersity and particle sizes are in the micrometer range. Later, flocs under the influence of polymer were analyzed using SEM and TEM to get insight into the morphology of flocs and the overall organization of polymer on top of particles. It was thus possible to calculate the compressive yield stress utilizing two sets of data—qualitative and quantitative—about the rate of sedimentation as well as the maximum packing in the presence and absence of a flocculant. Sample maximum packing was recorded as 44% and this value did not differ if PAM was added into the mix. The mean speed of sedimentation was found to be progressively dependent on the RPM of centrifugation and, across the kaolinite samples examined here, differed very slightly. The flocculated samples mean speed of sedimentation was reduced.

Then, if kaolinite behaved as hard spheres, we attempted to mimic maximum packing using validated strategies for doing so. The effectiveness of these methods to predict maximum packing given polydispersity was assessed. Due to the polydispersity of the particles produced by DLS, maximum packing may be generated via simulation and compared to experimental data. For both monodisperse and polydisperse hard spheres, simulation successfully determined the random close packing threshold and agreed with the data given in the literature. The results of the simulation and the maximum packing as determined empirically for the kaolinite samples in this investigation were in excellent agreement.

For future projects, it would be interesting to employ confocal laser scanning microscopy (CLSM) to view the sediment bed under the microscope after applying various centrifugal regimes; the results of CLSM, due to having greater resolution than LUMisizer, will probably be more accurate and can lead to obtaining further useful insights about the sedimented microstructure. In terms of simulation, it would be interesting to note that the capacity to model the right form of kaolinite particles will unquestionably lead to improved estimation for maximum packing through the development of less computationally costly techniques; however, the LS algorithm, “One of the simplest simulation tools”, becomes significantly computationally costly as the shapes of particles start to deviate from the spherical shape. The results of this study are also extendable to the production of liquid crystalline phases out of novel colloids such as cellulose nanocrystals [86] in the form of nanosheets or graphene papers [87] for energy storage applications.

A rough formula for the porosity of a binary mixture of non-spherical particles with intermediate size ratios was published by Yu et al. [88] in their study of packings of non-spherical particles, which was based on the Westman equation [89]. They introduced an “equivalent packing diameter” to approximate non-spherical particles to spherical ones for this purpose, and they then investigated how porosity changed with respect to the volume percentage and the size ratio of tiny and big particles. The empirical equation and experimental data showed good agreement. Therefore, using the same formula or school of thinking, the results of the simulation and experimental values examined here may simply be expanded from spherical particles to non-spherical particles.

## Figures and Tables

**Table 1:** Based on the results of Figure 6, an estimation of the maximum packing using LUMisizer profiles at a 4000-rpm centrifugation rotation speed is presented in the final column for all three different kaolinite samples.

Sample	Concentration [vol%]	Sediment Bed Length [mm]	Maximum Length [mm]	Length Difference [mm]	Volume of Particle Added [mm^3^]	Maximum Packing [-]
Sigma I	2.7	126.7 ± 0.5	130	3.3 ± 0.5	29.6	0.4 (0.35,0.46)
Sigma I	5.4	123.7 ± 0.5	130	6.3 ± 0.5	59.2	0.42 (0.41,0.48)
Sigma II	2.7	125.1 ± 0.5	130	4.9 ± 0.5	29.6	0.27 (0.25,0.31)
Sigma II	5.4	121.9 ± 0.3	130	8.1 ± 0.3	59.2	0.33 (0.32,0.34)
Riedel	2.7	126.1 ± 1.1	130	3.9 ± 0.3	29.6	0.34 (0.32,0.37)
Riedel	5.4	122.9 ± 0.5	130	7.1 ± 0.5	59.2	0.38 (0.35,0.41)

**Table 2:** Power law fits parameters associated with Figure 7, Figure 8 and Figure 9.

Sample	Applied Centrifugation [RPM]	Mean Speed of Sedimentation [%/s]
Sigma I	4000	0.25
Sigma II	4000	0.27
Riedel	4000	0.28
Sigma I	200	0.16
Sigma I	500	0.29
Sigma I	1000	0.33
Sigma I	4000	0.25
Sigma I + 7.5 ppm PAM	200	0.04
Sigma I + 15 ppm PAM	200	0.04
Sigma I + 7.5 ppm PAM	4000	0.03
Sigma I + 15 ppm PAM	4000	0.04

**Table 3:** Particle sizes were captured using LUMisizer software at fixed positions of 115, 120, and 125 mm along the height of the cell.

Condition [rpm]	Median, [nm]	Harmonic Mean, [nm]	Kaolinite Concentration [wt%]	PAM Concentration [ppm]
200	10100	10557	0.1	0
500	3957	2630	0.1	0
1000	2112	1300	0.1	0
4000	1302	893	0.1	0
200	1695	2135	0.1	7.5
4000	278.7	254	0.1	7.5
200	2719	2497	0.1	15
4000	262	241	0.1	15

**Table 4:** Simulation results are tabulated in instances with visual depictions as a function of polydispersity and compression rate.

Compression Rate	Polydispersity [-]	Force-Biased (FB)	LS	LSGD	Visual Depiction
1.00 × 10^−1^	σ=0	2.89 × 10^−1^	6.18 × 10^−1^	6.42 × 10^−1^	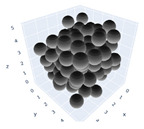
1.00 × 10^−2^	σ=0	5.07 × 10^−1^	6.41 × 10^−1^	6.41 × 10^−1^	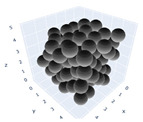
1.00 × 10^−3^	σ=0	5.85 × 10^−1^	6.45 × 10^−1^	6.45 × 10^−1^	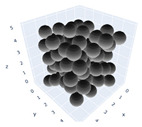
1.00 × 10^−4^	σ=0	6.29 × 10^−1^	6.34 × 10^−1^	6.34 × 10^−1^	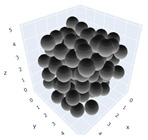
1.00 × 10^−5^	σ=0	6.47 × 10^−1^	6.48 × 10^−1^	6.48 × 10^−1^	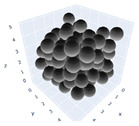
1.00 × 10^−1^	σ=0.1	3.13 × 10^−1^	6.01 × 10^−1^	6.29 × 10^−1^	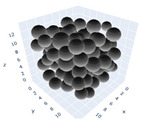
1.00 × 10^−2^	σ=0.1	5.10 × 10^−1^	6.29 × 10^−1^	6.37 × 10^−1^	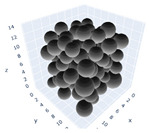
1.00 × 10^−3^	σ=0.1	5.99 × 10^−1^	6.43 × 10^−1^	6.43 × 10^−1^	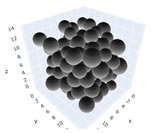
1.00 × 10^−4^	σ=0.1	6.32 × 10^−1^	6.34 × 10^−1^	6.34 × 10^−1^	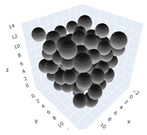
1.00 × 10^−5^	σ=0.1	6.38 × 10^−1^	6.39 × 10^−1^	6.39 × 10^−1^	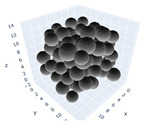
1.00 × 10^−1^	σ=0.2	2.99 × 10^−1^	6.29 × 10^−1^	6.37 × 10^−1^	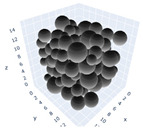
1.00 × 10^−2^	σ=0.2	4.70 × 10^−1^	6.48 × 10^−1^	6.50 × 10^−1^	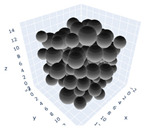
1.00 × 10^−3^	σ=0.2	6.12 × 10^−1^	6.46 × 10^−1^	6.46 × 10^−1^	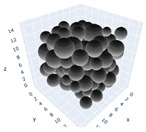
1.00 × 10^−4^	σ=0.2	6.44 × 10^−1^	6.53 × 10^−1^	6.53 × 10^−1^	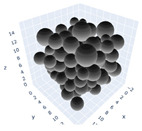
1.00 × 10^−5^	σ=0.2	6.47 × 10^−1^	6.47 × 10^−1^	6.47 × 10^−1^	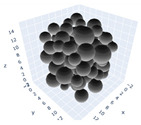
1.00 × 10^−1^	σ=0.3	3.12 × 10^−1^	6.19 × 10^−1^	6.65 × 10^−1^	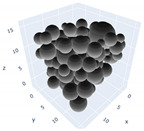
1.00 × 10^−2^	σ=0.3	5.24 × 10^−1^	6.45 × 10^−1^	6.50 × 10^−1^	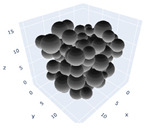
1.00 × 10^−3^	σ=0.3	6.08 × 10^−1^	6.61 × 10^−1^	6.61 × 10^−1^	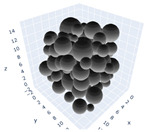
1.00 × 10^−4^	σ=0.3	6.54 × 10^−1^	6.68 × 10^−1^	6.68 × 10^−1^	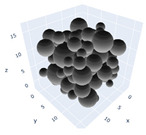
1.00 × 10^−5^	σ=0.3	6.54 × 10^−1^	6.55 × 10^−1^	6.55 × 10^−1^	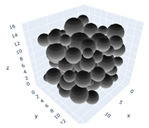
1.00 × 10^−1^	σ=0.4	3.17 × 10^−1^	6.19 × 10^−1^	6.72 × 10^−1^	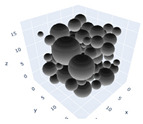
1.00 × 10^−2^	σ=0.4	5.11 × 10^−1^	6.67 × 10^−1^	6.71 × 10^−1^	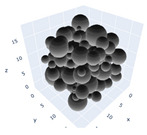
1.00 × 10^−3^	σ=0.4	6.19 × 10^−1^	6.78 × 10^−1^	6.78 × 10^−1^	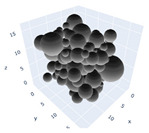
1.00 × 10^−4^	σ=0.4	6.59 × 10^−1^	6.86 × 10^−1^	6.86 × 10^−1^	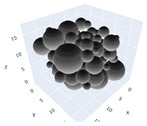
1.00 × 10^−5^	σ=0.4	6.69 × 10^−1^	6.70 × 10^−1^	6.70 × 10^−1^	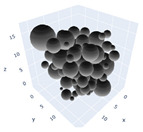

**Table 5:** Estimation of compressive yield stress-based data presented earlier.

Sample	Concentration [vol%]	Sediment Bed Length [mm]	Maximum Length [mm]	Length Difference [mm]	Compressive Yield Stress [kPa]
Sigma I	2.7	126.7 ± 0.5	130	3.3 ± 0.5	49
Sigma I	5.4	123.7 ± 0.5	130	6.3 ± 0.5	94
Sigma II	2.7	125.1 ± 0.5	130	4.9 ± 0.5	48
Sigma II	5.4	121.9 ± 0.3	130	8.1 ± 0.3	92
Riedel	2.7	126.1 ± 1.1	130	3.9 ± 0.3	48
Riedel	5.4	122.9 ± 0.5	130	7.1 ± 0.5	93

## Data Availability

Not applicable.
